# Design and Characterization of Inhibitors of Cell-Mediated Degradation of APOBEC3G That Decrease HIV-1 Infectivity

**DOI:** 10.3390/v17040514

**Published:** 2025-04-01

**Authors:** Aubrey M. Sawyer, Cristina C. Vaca, Neha Malik, Isabelle Clerc, Joshua Craft, Hannah Hudson, Gaël K. Scholtés, Gary E. Schiltz, Meejeon Roh, Chisu Song, Richard T. D’Aquila

**Affiliations:** 1 Department of Medicine, Division of Infectious Diseases, Northwestern University Feinberg School of Medicine, Chicago, IL 60611, USAhannah.hudson@northwestern.edu (H.H.);; 2 Department of Chemistry, Northwestern University Weinberg College of Arts and Sciences, Evanston, IL 60208, USA; 3 Robert H. Lurie Comprehensive Cancer Center of Northwestern University, Chicago, IL 60611, USA; 4 Department of Pharmacology, Northwestern University Feinberg School of Medicine, Chicago, IL 60611, USA; 5 Department of Surgery, University of Chicago Medicine, Chicago, IL 60637, USA

**Keywords:** APOBEC3G, APOBEC3F, Vif, HIV, HIV-1, HIV cure research, HIV infectivity

## Abstract

The cytoplasmic human Apolipoprotein B mRNA-editing enzyme catalytic polypeptide-like 3 (APOBEC3 or A3) cytidine deaminases G and F (A3G and A3F) can block the spread of human immunodeficiency virus (HIV). HIV counteracts this cell-intrinsic defense through a viral protein called viral infectivity factor (Vif). Vif causes proteasomal degradation of A3G and A3F proteins (A3G/F) in HIV-producing cells to ensure infectivity of virions subsequently released from these cells. Here, we optimized a lead compound reported previously to boost cellular levels of A3G. The modified analogs designed, synthesized, and evaluated here inhibit cell-mediated post-translational degradation of A3G/F, whether Vif is present or not. This increases A3G/F incorporation into Vif-positive virions to decrease viral infectivity. The compounds and processes described here can facilitate the development of new anti-HIV therapeutics whose host-targeted effect may not be evaded by resistance-conferring mutations in HIV Vif.

## 1. Introduction

There are seven members of the apolipoprotein B mRNA-editing enzyme catalytic polypeptide-like 3 (APOBEC3; A3) family of cellular cytidine deaminases in humans: A3A, -B, -C, -DE, -F, -G, and -H [1,2,3]. Their conserved cytidine deaminase catalytic function introduces mutations in different single-stranded (ss) DNA substrates, depending on whether the A3s are localized to the nucleus or cytoplasm [4]. Nuclear-localized A3s (A3A, A3B, and one A3H haplotype) are a source of chromosomal DNA mutagenesis in cancer cells that can promote oncogenesis [5,6,7,8,9,10,11,12,13]. A3 enzymes that are predominately localized to the cytoplasm (A3G, A3F, A3D, some A3H haplotypes) can be incorporated into retroviruses during virion assembly in select circumstances [14,15,16,17,18]. If incorporated into the virion, A3s hypermutate the ssDNA intermediate during reverse transcription in the next cell they infect [18,19,20]. This introduces premature stop codons into the resulting proviruses that can preclude production of one or more essential virus proteins, restricting subsequent rounds of infection. Virion-incorporated A3s can also inhibit HIV replication through cytidine deaminase independent mechanisms [21,22].

Retroviruses evolved a mechanism to counteract A3-mediated restriction of their infectivity by post-translationally degrading the A3s in virus-producing cells. HIV encodes virion infectivity factor (Vif), which recruits the cytoplasmic A3 proteins to a specific Cullin-RING E3 ubiquitin ligase (CRL) complex [23,24,25,26]. This CRL complex is co-opted by Vif and polyubiquitinates the cytoplasmic A3s. This modification directs the A3s for proteasomal degradation prior to assembly and thereby efficiently prevents their incorporation into budding virions. Although these A3s can only completely block Vif-null HIV spread, some inhibition of Vif+ HIV spread was seen ex vivo in a comparison of cells with higher, relative to lower, endogenous levels of cytoplasmic A3s prior to Vif+ infection [27,28]. Small molecules were reported previously to antagonize HIV Vif but have not advanced toward development [29,30,31].

**Figure 1:**
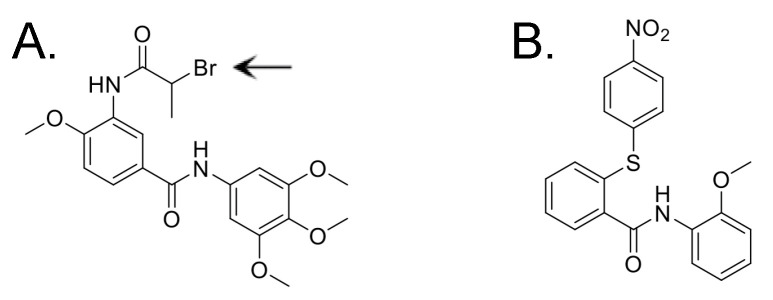
Structural formulas of compounds previously identified to inhibit Vif-mediated A3G degradation. Methyl groups indicated by a line. (**A**) IMB-26. Arrow indicates R_2_ site. (**B**) RN-18.

Here, we optimize a lead candidate reported previously to boost A3G levels in the presence of Vif (IMB-26, Figure 1A) [29]. We demonstrate that new analogs with modifications at their R_2_ site (Figure 1A) increase cellular and viral levels of A3G and A3F proteins and decrease Vif+ HIV infectivity ex vivo whether Vif is present or not. We also demonstrate that these novel compounds interfere with entirely cell-encoded processes. This suggests the hypothesis that these new compounds may be less vulnerable to escape due to virus mutation than a previously reported, structurally different agent that quickly selected for a resistance-conferring mutation in Vif in vitro (RN-18, Figure 1B) [30,31]. The novel compounds described here, and the processes used to evaluate them, can help develop agents intended to increase cellular A3G and A3F levels prior to Vif expression, with the goal of diminishing Vif+ viral spread after stopping antiretroviral therapy (ART). Such agents could be an adjunct in a broader strategy to cure HIV.

## 2. Materials and Methods

### 2.1. Cell Lines and Culture Conditions

293T, CEM, and H9 cells were obtained from ATCC. TZM-bl cells were obtained from the NIH HIV Reagent Program (part of BEI Resources Repository). The TZM-bl indicator cell line, used for HIV infectivity assays, is a genetically engineered HeLa cell clone with an integrated Tat-responsive firefly luciferase transcribed from an HIV-1 long terminal repeat. 293T and TZM-bl cells were cultivated in DMEM medium containing 10% fetal bovine serum (FBS), 4.5 g/liter glucose, L-glutamine, sodium pyruvate, 50 IU/mL penicillin, and 50 μg/mL streptomycin. H9 and CEM cells were maintained in RPMI medium plus 10% FBS, 50 IU/mL penicillin, and 50 μg/mL streptomycin. All cells were maintained at 37 °C and 5% CO_2_.

### 2.2. Isolation and Activation of Primary CD4+ T Cells

Whole blood from leukoreduction chambers after Trima apheresis (Cytek, San Diego, CA, USA) from unidentifiable healthy human donors underwent Ficoll centrifugation to isolate peripheral blood mononuclear cells (PBMCs). PBMCs were used fresh or after cryopreservation in liquid nitrogen at 50 million cells per 1 mL of 90% FBS with 10% DMSO. CD4+ cells were isolated from PBMCs using negative selection via an EasySep Human CD4+ T-cell isolation kit (Cytek, per manufacturer’s instructions). Isolated CD4+ T cells were activated by plate-bound α-CD3 (Cytek) and α-CD28 (Cytek) in complete RPMI media with 10% FBS and 50 µg/mL penicillin-streptomycin at 37 °C and 5% CO_2_ and maintained in that media.

### 2.3. Plasmids

The Vif+ (wild-type) full-length infectious HIV-1 proviral genome (NL4.3) expression plasmid used here [32] and the isogenic pNL4.3 Vif-null mutant derived from it were described previously [16]. pcDNA3.1 was used as empty control plasmid across transient transfection experiments, including for the two NL4.3 constructs. A3G and A3F expression plasmids with and without an HA tag were also described previously [15,16]. pMSCV-I/Puro retroviral expression vector was constructed by replacing the neomycin resistance gene using EcoRI-ClaI with IRES-Puro genes from IRES-puro-GFP plasmid (Addgene plasmid #16616, a gift from Bert Vogelstein) [33]. An A3G-luciferase (A3G-Luc) expression plasmid was constructed by three-way ligation of A3G and luciferase coding sequences into a pMSCV-I/Puro vector. A full-length A3G open-reading frame was constructed by PCR with *Age*I and *Not*I sites at the 5′ and 3′ ends, respectively, from a pcDNA-A3G template. A full-length Luc fragment with *Not*I and *EcoR*I sites at the 5′ and 3′ ends, respectively, was generated by PCR from the pcDNA-Luc plasmid. The pMSCV-I/Puro plasmid digested with *Age*I and *Eco*RI, the A3G fragment digested with *Age*I/*Not*I, and the Luc fragment digested with *Not*I/*EcoR*I were ligated to generate pMSCV-I/Puro-A3G-Luc plasmid expressing A3G-Luc fusion protein from a retroviral 5′ LTR (along with a packaging signal and 3′ LTR). In parallel, a control pMSCV-I/Puro Luc plasmid was constructed without A3G coding sequences.

A Vif-expressing lentiviral vector was constructed by inserting the Vif coding sequences into *Nhe*I and *Age*I sites of a FUGW lentiviral vector [34]. The Vif fragment was generated by PCR from the NL4.3 Vif gene (pcDNA-hVif) using appropriate oligonucleotide primers for *Nhe*I and *Age*I sites [35].

Plasmids expressing unfused A3G and A3F were also constructed. A pMSCV-I/Puro-A3G expression vector was constructed by ligating A3G coding sequences between *EcoR*I and *Not*I sites. The A3G fragment was generated by PCR from pcDNA-A3G using oligonucleotide primers with *EcoR*I and *Not*I sites. The corresponding pMSCV-I/Puro-A3F was constructed by inserting A3F coding sequences between *Age*I and *Not*I sites. The A3F fragment was generated by PCR from pcDNA-A3F using oligonucleotide primers for *Age*I and *Not*I. Expression plasmids for pVHL were described in [36].

The primer sequences used for the construction of the pMSCV-I/Puro-A3G-Luc, FUGW-Vif, pMSCV-I/Puro-A3G, pMSCV-I/Puro-A3F, and corresponding controls are available on request.

### 2.4. Construction of Cell Lines Stably Expressing Proteins of Interest

To generate clonal cell lines stably expressing the A3G-Luc fusion protein at different levels, 293T cells (which lack endogenous expression of A3s) were co-transfected with pMSCV-I/Puro-A3G-Luc, pCL-Ampho (Novus Biologicals, Centennial, CO, USA) as a murine retroviral packaging plasmid, as well as pCMV-VSV-G (Addgene plasmid #8454, a gift from Bob Weinberg) [37] encoding the VSV envelope. The resulting pseudotyped viral particles were transduced into 293T cells, followed by selection for puromycin resistance.

To generate cell lines stably expressing both A3G-Luc and Vif proteins, the lines expressing A3G-Luc at different levels were each transduced with pseudotyped viral particles generated by co-transfecting 293T cells with a lentiviral vector encoding HIV-1 Vif (FUGW-Vif), pSAX2 (Addgene plasmid #12260, a gift from Didier Trono) as a lentiviral packaging plasmid, as well as pCMV-VSV-G. A control cell line lacking Vif was generated by infecting with viral particles produced from a co-transfection of the control FUGW, pSAX2, and pCMV-VSV-G, followed by selection in puromycin. Since the FUGW vector encodes the yellow fluorescent protein (YFP) reporter gene, YFP-positive cells were sorted using FACS for both the Vif-expressing and control lines. After sorting for YFP, the presence and absence of the expression of Vif, respectively, was confirmed by immunoblotting.

To generate stable cell lines expressing unfused A3G, A3F, or control, 293T cells were transduced with viral particles generated by co-transfecting either pMSCV-I/Puro-A3G, pMSCV-I/Puro-A3F, or pMSCV-I/Puro (lacking either A3 coding sequence), along with packaging and envelope constructs (pCL-Ampho and pCMV-VSV-G). Transduced cells were selected in puromycin (0.75 μg/mL). Stable expression of A3G, A3F, or neither was confirmed by immunoblotting using antibodies against A3G or A3F in the puromycin-selected 293T I/Puro G, 293T I/Puro F, or 293T I/Puro (empty vector control) cells.

### 2.5. Synthesis of Compounds

Synthetic routes for the analogs of IMB-26 (Figure 1A) studied here are shown in Supplemental Figure S1A–C. Amide formation between 4-methoxy-3-nitrobenzoic acid (compound 1) and various anilines and benzylamines took place using standard HOBt/EDC coupling conditions (Supplementary Figure S1A). Reduction of the 3-nitro group of compound 2 using SnCl_2_ in refluxing ethanol produced good yields of amines (3). Finally, acylation of the amine using a variety of acid chlorides efficiently provided the desired compounds (4). Supplementary Figure S1B shows the route for making derivatives of R_1_-anlines (compound 7). In this sequence, methyl 4-methoxy-3-nitrobenzoate was first reduced with SnCl_2_ and acylated to provide intermediate amide 6. After hydrolysis with NaOH, amide coupling using HOBt/EDC provided the R_2_ analogs (7). Mono-amide 6 was methylated using NaH and methyl iodide to provide compound 8 in good yield. Hydrolysis and amide formation provided bis-amide 9. Finally, several ether analogs were prepared, as shown in Supplemental Figure S1C. The ethers were formed by treating methyl 4-hydroxy-3-nitrobenzoate with the alkyl halides to provide ether 11. Reduction of the nitro group and acylation afforded amides 12. Hydrolysis and amide formation yielded the desired bis-amide ether analogs 13.

### 2.6. Initial Evaluation of Compounds

Effects of new compounds synthesized here on A3G-Luc activity were evaluated relative to control (DMSO). 293T cells stably expressing A3G-Luc or A3G-Luc/Vif were seeded at 25,000 cells per well in 96-well plates. After 24 h, culture medium was replaced with 100 μL of new medium containing either DMSO or one of the novel compounds (10 μM concentration). After 24, 48, or 72 h of further incubation with the compound, the culture medium was removed from each well and replaced with 100 μL of Britelite Plus luciferase assay substrate (PerkinElmer, Shelton, CT, USA). Following 5 min of incubation at room temperature, 75 μL of each cell lysate was transferred to a 96-well OptiPlate 96 (PerkinElmer), and luminescence was measured in a VICTOR X2 Multilabel Reader (PerkinElmer). Relative activity of compounds for boosting A3G-Luc was calculated by normalizing their relative luminescence units (RLUs) readouts to that of DMSO-treated cells expressing A3G-Luc or A3G-Luc/Vif.

### 2.7. Antibodies

The following antibodies were obtained from the NIH HIV Reagent Program (now part of BEI Resources Repository): an anti-APOBEC3F(C18) polyclonal antibody, which recognizes an epitope at the C-terminus of A3F; anti-APOBEC3G polyclonal antibody (C17); and an anti-Vif polyclonal antibody. Anti-β-actin monoclonal antibody (clone AC-74) and anti-GAPDH monoclonal antibody were purchased from Sigma. Anti-Lamin B1 was from Proteintech. The anti-HA rabbit polyclonal antibody was from US Biologicals, the anti-FLAG antibody was from Sigma, and the anti-p24 monoclonal antibody 183-H12–5C was from the Tennessee Center for AIDS Research Virology Core.

### 2.8. Immunoblotting of Cells and Viruses for A3s

Immunoblotting of cell lysates and viral pellets was carried out at 24 h after transfections using linear polyethylenimine (PEI; 25 kDa; Polysciences, Inc., Warrington, PA, USA), as described [38]. For detection of cellular levels of A3F and A3G proteins, 293T cells were plated at a density of 7.5 × 10^5^ cells/well in a 6-well culture plate 24 h prior to transfection with either 1 ug of pcDNA-A3F or 500 ng of pcDNA-A3G. The transfected cells were lysed in 250 μL of cell lysis buffer (1× Dulbecco’s Phosphate-buffered Saline (Mediatech, Inc., Manassas, VA, USA), 1 mM Na_2_EDTA, 0.5% Triton X-100 (*v*/*v*), and complete mini protease inhibitor mixture without Na_2_EDTA (Roche)) and centrifuged at 10,000× *g* for 5 min at 4 °C.

To assess the effect of compounds on HIV infectivity, 293T cells were co-transfected transiently with NL4.3 (1.5 μg), as well as A3F (150 μg) or A3G (150 μg) expression plasmids. Additionally, 293T cells stably expressing either A3F or A3G were transiently transfected with NL4.3 (1.5 μg). At 24 h post-transfection, fresh media containing either NU compounds or DMSO were added. After an additional 24 h, viral particles were pelleted through a 20% sucrose cushion at 32,000 rpm for 2 h, followed by immunoblotting.

Cell lysates or viral pellets were combined with 25 μL of 2× SDS-protein sample buffer (100 mM Tris-HCl, pH 6.8, 4 mM Na_2_EDTA, 4% SDS, 4% 2-mercaptoethanol, 20% glycerol, 0.1% bromphenol blue), heated at 100 °C for 5 min, and analyzed by electrophoresis through a 4–12% SDS-polyacrylamide gel. After electrophoresis, separated proteins were transferred to an Immobilon-P membrane (MilliporeSigma, St. Louis, MO, USA) and processed for Western blot analysis using protein-specific antibodies with chemiluminescent detection. When fluorescently labeled secondary antibodies were used, immunoblots were quantified using either ImageJ or the Odyssey Clx imaging system with Empiria Studio (LiCor, Lincoln, NE, USA).

### 2.9. Viral Infectivity Assay

TZM-bl reporter cells were plated at a density of 10,000 cells/well in a 96-well plate 24 h prior to infection and incubated at 37 °C (5% CO_2_). On the day of infection, the culture medium was removed, and the cells were inoculated in triplicate with 100 μL of 2-fold serial dilutions of viral supernatants after normalization for HIV-1 p24 by enzyme-linked immunosorbent assay (ELISA) (Revvity, Boston, MA, USA) in culture medium containing 20 μg/mL DEAE-dextran and incubated at 37 °C (5% CO_2_). After 24 h of incubation, the culture medium was removed from each well, and viral infectivity was measured via luciferase activity, as described above. Relative infectivity was calculated by normalizing RLU to that of the viral particles from DMSO-treated cells expressing A3G or A3F.

### 2.10. Protein Stability Assay

293T cells stably expressing A3G-Luc fusion protein (25,000 cells/well) were plated in 96-well plates. The luciferase activity of 293T cells expressing A3G-Luc was measured 24 h after cycloheximide addition. 293T cells were plated at a density of 1 million cells/well in 6-well plates. One day after plating, the cells were transfected with A3G plasmid (1.5 μg). One day post-transfection, the culture media was removed, and cycloheximide (50 μg/mL) (MilliporeSigma, St. Louis, MO, USA) alone or cycloheximide with compounds (10 μM) was added to the medium. 293T cells transfected with A3G were harvested 24 h after cycloheximide addition, and the effect of compounds on A3G protein stability was analyzed by immunoblotting using anti-A3G antibody.

### 2.11. HIV-1 Infections

H9 cells (4 × 10^6^ cells) were plated in a 6-well plate and incubated with 3 ng of Vif+ NL4.3 or Vif-null NL4.3. Plates were sealed and spun at 1200× *g* for 2 h at room temperature. Spinoculated cells were incubated in 6-well plates at 37 °C 5% CO_2_ overnight before transferring to T25 tissue culture flasks with NU52 or DMSO as control. Cells and supernatant from each culture flask were collected at 2 days post-infection for downstream applications.

## 3. Results

### 3.1. Selection of Cell Lines Stably Expressing A3G-Luciferase (A3G-Luc)

**Figure 2:**
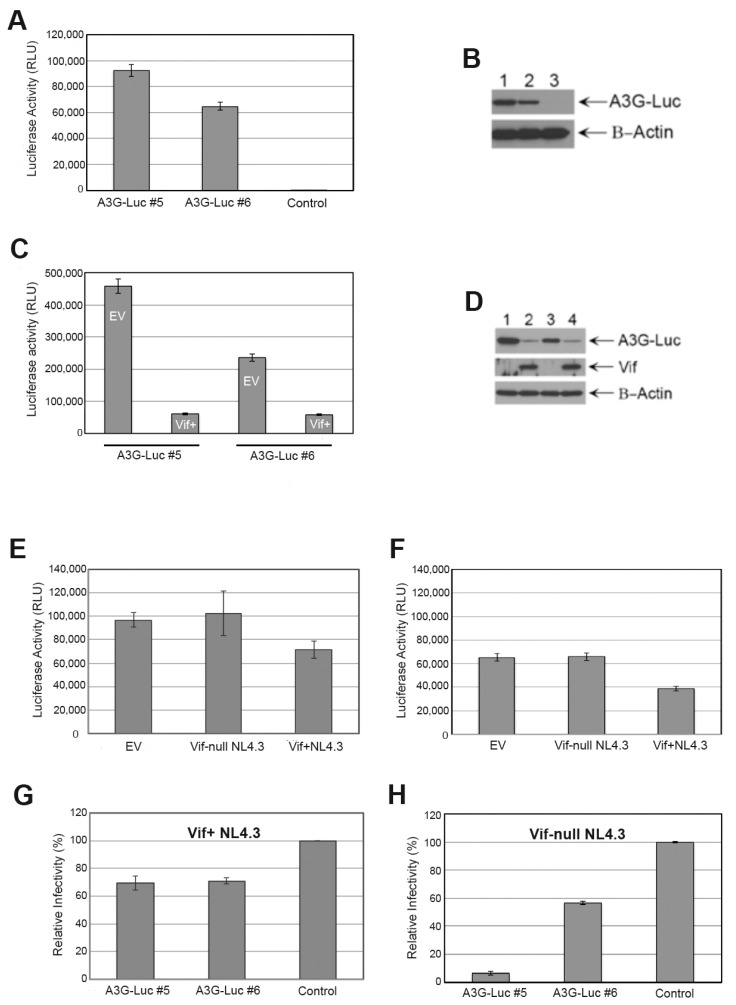
Characterization of puromycin-resistant 293T-cell lines stably expressing A3G-Luc and Vif/A3G-Luc. (**A**) Relative luciferase activities (RLUs) of two different clones (#5 and #6) transduced by pseudotyped viral particles to stably express different levels of A3G-Luc. A similarly transduced control clonal line lacks A3G-Luc expression. (**B**) Immunoblots of these three clones probed with an anti-A3G antibody are shown. Lane 1 is A3G-Luc #5. Lane 2 is A3G-Luc #6. Lane 3 is the control line. (**C**) RLU of clone #5 or clone #6, each with or without stable expression of Vif using the FUGW-Vif vector (indicated as Vif+) and the corresponding empty vector (indicated as EV) control. (**D**) Immunoblots for A3G, Vif, and β-actin proteins from C are shown. Lane 1 is EV/A3G-Luc #5. Lane 2 is Vif/A3G-Luc #5. Lane 3 is EV/A3G-Luc #6. Lane 4 is Vif/A3G-Luc #6. (**E**) Luciferase activities produced by A3G-Luc cell clone #5 following transfection with the EV control, the Vif-null NL4.3 plasmid, or the Vif+ NL4.3 plasmid are shown. (**F**) Luciferase activities produced by A3G-Luc cell clone #6 following transfection with the EV control, the Vif-null NL4.3 plasmid, or the Vif+ NL4.3 plasmid are shown. (**G**) Relative infectivity of viral particles produced from transfection of Vif+ NL4.3 expression plasmid (indicated as Vif+) into A3G-Luc cell clones #5 and 6, as well as the control line, are shown. Supernatants of Vif+ NL4.3-transfected cultures of each cell clone were normalized by p24 value prior to infection of TZM-bl cells to ensure the same viral input. RLUs were read 24 h after infection of TZM-bl cells. (**H**) Relative infectivity of viral particles produced from transfection of Vif-null NL4.3 expression plasmid (indicated as Vif-null) into A3G-Luc cell clones #5 and 6, as well as the control line, are shown. Infectivity was assessed as in G.

Ten clonal puromycin-resistant 293T cell lines stably expressing A3G-Luc were generated, as described in Materials and Methods. Two A3G-Luc expressing clones were chosen because of their different luciferase activities (Figure 2A) and A3G protein levels detected on immunoblots (Figure 2B). A3G-Luc clone #5 displays the highest levels of Luc activity and A3G immunoreactivity (Figure 2A,B). A3G-Luc clone #6 has relatively lower levels of both luciferase activity and A3G immunoreactivity (Figure 2A,B). One puromycin-resistant, retrovirus-transduced clone lacked any luciferase activity or A3G expression and served as an A3G-Luc negative control (control in Figure 2A,B,G,H).

The ability of Vif to degrade the different levels of the A3G-Luc fusion protein in A3G-Luc clones #5 and #6 was tested. We found that expression of functional Vif from the FUGW-Vif lentiviral vector decreased A3G-Luc luciferase activity (Figure 2C) and anti-A3G antibody detection on immunoblots (lanes 2 and 4 in Figure 2D), relative to the FUGW empty vector (EV) control. Stable expression of Vif decreased both luciferase activity and the A3G-Luc protein to similarly low levels in each of the 2 A3G-Luc clones (Figure 2C,D). We also evaluated Vif function when it was expressed from transfected Vif+ NL4.3. Two days after transfection of Vif+ NL4.3, luciferase activity was reduced in A3G-Luc clones #5 (Figure 2E) and #6 (Figure 2F), relative to both the empty vector (EV, pcDNA) control and Vif-null NL4.3.

In addition, we tested if the A3G-Luc fusion protein packaged into virions retained the ability to restrict infectivity of the virus in TZM-bl cells. After each A3G-Luc clone was transfected with Vif+ NL4.3, there was some reduction in relative infectivity, relative to the transfected A3G-Luc negative control cell supernatant (Figure 2G). After Vif-null NL4.3 transfection, greater reductions were seen (Figure 2H). Infectivity of Vif-null virus from the supernatant of A3G-Luc clone #5 culture was decreased more than that of Vif-null virus from the A3G-Luc clone #6 culture supernatant, as normalized to the infectivity of the supernatant from the transfected A3G-Luc negative control cells (Figure 2H).

These results indicate that these A3G-Luc and Vif/A3G-Luc cell lines can be used to screen analogs synthesized here for the ability to increase cell and virion A3G-Luc levels, as well as to restrict virion infectivity, in the absence or presence of Vif.

### 3.2. Compounds Modified from IMB-26 Boost A3G and A3F Regardless of Vif Expression

The cell lines above were used to study IMB-26 and to start the evaluation of modifications of it that were synthesized here (listed in Supplementary Table S1). IMB-26 was previously described to bind to A3G and prevent its degradation by Vif (Figure 1A and first listing in Supplementary Table S1) [29]. A3G-Luc or Vif/A3G-Luc cells (both #5 and #6 clones) were plated, and 24 h later, each of these analogs, or DMSO as a control, was added to the culture media. Luciferase activity (in relative luminescence units, RLUs) was measured 24, 48, and 72 h after addition of the compound or control. In our hands, IMB-26 did not increase A3G-Luc activity, relative to the control, in either the presence or absence of Vif (Supplementary Table S1 and Figure S2). IMB-26 was also cytotoxic. This is consistent with an earlier report showing the cytotoxicity of related analogs with either iodo- or bromo-modifications at the R_2_ site [39]. Two of three analogs of IMB-26 with bromine at the R_2_ site also did not increase A3G-Luc activity in the presence or absence of Vif; one of them did increase it (halogenated compounds in Supplementary Table S1).

The cytotoxicity observed here led us to next evaluate analogs with different replacements for a bromine at the R_2_ site. Most of these des-bromo analogs increased A3G-Luc activity both in the presence and absence of Vif, although some increased A3G-Luc activity only in the absence of Vif (des-bromo compounds are the remainder listed in Supplementary Table S1 following the halogenated compounds). Exploration of the R_2_ site with a range of amides showed larger increases in A3G-Luc activity with small non-aromatic amides. Groups that contained heteroatoms were also well-tolerated and produced robust increases in A3G-Luc activity in both the presence and absence of Vif (Supplementary Table S1). Replacements at the R_2_ site with carbamates, sulfonamide, or ureas also resulted in compounds that produced robust increases in A3G-Luc activity in both the presence and absence of Vif (Supplementary Table S1). Several ether analogs at the R_2_ site were synthesized; both the ethyl and methoxyethyl ether analogs produced similar A3G-Luc-boosting effects as their methyl ether counterparts in both the presence and absence of Vif (Supplementary Table S1).

**Figure 3:**
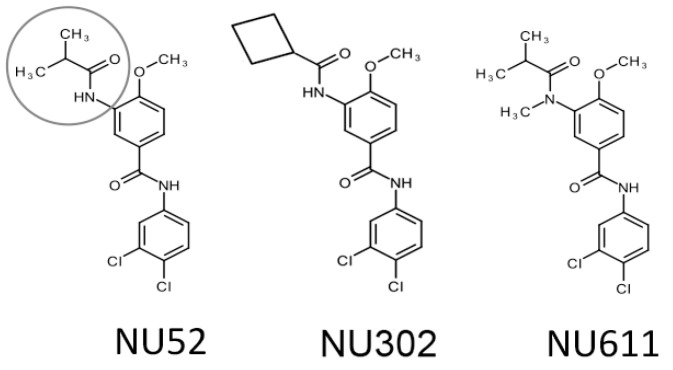
Structural formulas of compounds selected for further study. The R_2_ site is circled in NU-52. Note that methyl groups are shown here, rather than being depicted only as lines, as in Figure 1 and Supplementary Table S1.

Among the initial non-halogenated analogs tested, 10 uM of NU-52 resulted in A3G-Luc activity that was 178% of the DMSO control; Vif/A3G-Luc activity was 135% of the DMSO control (Supplementary Table S1; Figure 3 highlights the R_2_ position). Additional variants were synthesized and tested for effects on A3G-Luc. At 10 μM, NU-302 (Figure 3) showed A3G-Luc activity that was 196% of the DMSO control and Vif/A3G-Luc activity that was 167% of the DMSO control. NU-611 (Figure 3) was less cytotoxic than either NU-52 or NU-302, with no cell death observed even up to 25 μM. At 10 μM, NU-611 had A3G-Luc activity that was 197% of the DMSO control and Vif/A3G-Luc activity that was 175% of the DMSO control.

**Figure 4:**
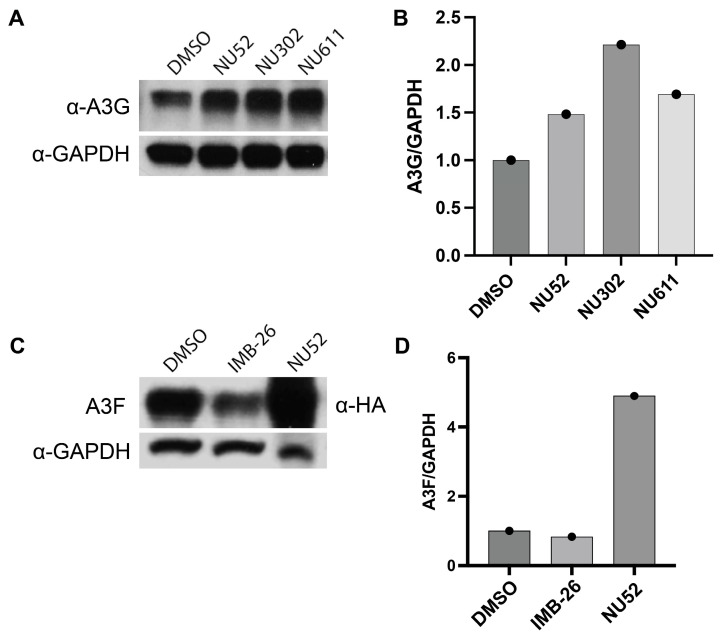
NU compounds boost A3G protein levels in uninfected CD4+ T cells and A3F protein levels in A3F-transfected 293T cells. (**A**) Immunoblot shows resting, uninfected CD4+ T cells treated with NU-52, -302, and -611 (10 μM each). (**B**) Quantification of band densities in A. (**C**) Immunoblot shows 293T cells transiently transfected with A3F expression plasmid and treated with DMSO, IMB-26, or NU-52. (**D**) Quantification of band densities in C.

We then treated uninfected primary CD4+ T cells with each of these three NU compounds. A3G levels were increased, as shown by immunoblots after exposure of CD4+ T cells to NU-52, -302, or -611, each at 10 μM, for 24 h (Figure 4A,B). We also found that 24 h exposure to NU-52 after either A3G or A3F were transiently transfected into 293T cells increased A3G and A3F levels on immunoblots, relative to DMSO (Figure 4B,C; Supplementary Figure S2). IMB-26 did not increase A3F (Figure 4C,D) or A3G (Supplementary Figure S2).

### 3.3. NU Compounds Increase Vif+ HIV Virion A3 Content and Reduce Vif+ HIV Virion Infectivity

We studied A3G or A3F content, as well as the relative infectivity, of Vif+ virions produced from cells treated with NU-52 or NU-302. 293T cells were co-transfected with the Vif+ HIV-1 proviral clone, NL4.3, along with either A3G or A3F expression plasmids. NU-52 (10 μM), NU-302 (10 μM), or DMSO were added 24 h after co-transfection. One day later, viral supernatants were collected and normalized by HIV p24 ELISA. Treatment with either of the two NU compounds did not affect amounts of viral particles produced in the transfected cells. 

**Figure 5:**
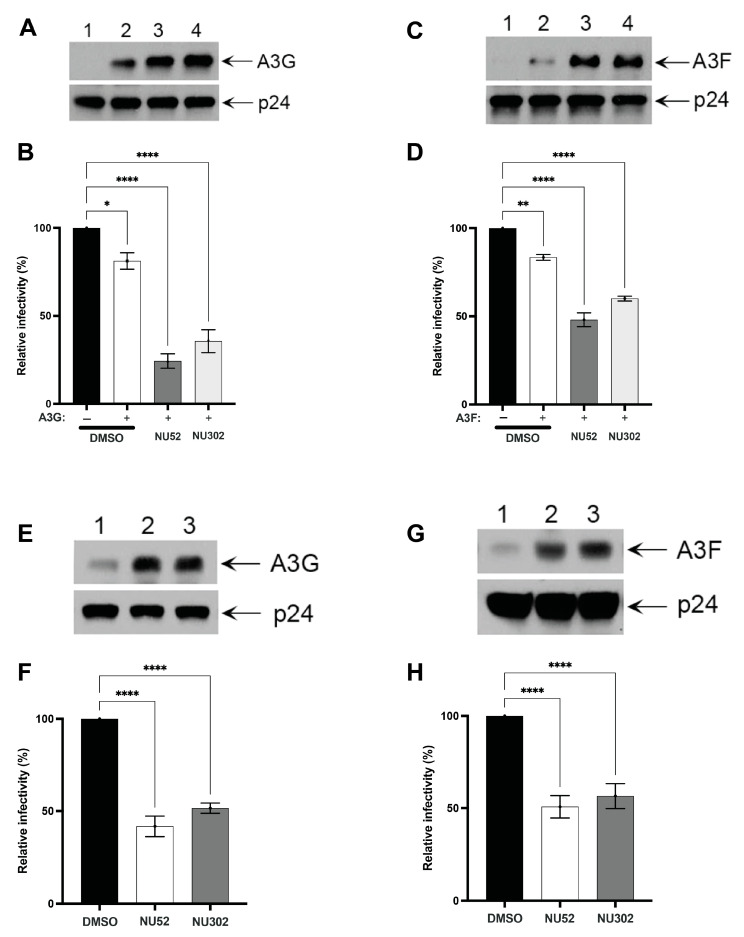
NU compounds increase incorporation of A3G or A3F into Vif+ virions and decrease Vif+ virion infectivity. (**A**) Vif+ NL4.3 was co-transfected with an A3G expression plasmid into 293T cells. DMSO or NU compounds (10 μM each) were added 24 h after transfection. One day later, immunoblots of A3G and p24 of pelleted supernatant viral particles were performed from cultures transfected with Vif+ NL4.3 alone (Lane 1) or co-transfected with both Vif+ NL4.3 and A3G followed by treatment with either DMSO (Lane 2), NU-52 (Lane 3), or NU-302 (Lane 4). (**B**) Relative infectivity of supernatant viruses from the different conditions described in A was determined. Vif+ HIV infectivity was measured by luciferase activity after infection of TZM-bl cells. (**C**) Immunoblots of A3F and p24 in pelleted supernatant viral particles from 293T cells transfected with Vif+ NL4.3 alone (Lane 1) or co-transfected with both Vif+ NL4-3 and A3F followed by treatment with either DMSO (Lane 2), NU-52 (Lane 3), or NU-302 (Lane 4) are shown. (**D**) Relative infectivity of supernatant viruses from 293T cells from the different conditions described in C was determined. Vif+ HIV infectivity was measured as in B. (**E**) Immunoblots of A3G and p24 in pelleted supernatant viral particles from 293T cells stably expressing A3G that were transiently transfected with Vif+ NL4.3 and treated with either DMSO (Lane 1), NU-52 (Lane 2), or NU-302 (Lane 3) are shown. (**F**) Relative infectivity of supernatant viruses from 293T cells stably expressing A3G from the different conditions described in E was determined. (**G**) Immunoblots of A3F and p24 in pelleted supernatant viral particles from 293T cells stably expressing A3F that were transiently transfected with Vif+ NL4.3 and treated with either DMSO (Lane 1), NU-52 (Lane 2), or NU-302 (Lane 3) are shown. (**H**) Relative infectivity of supernatant viruses from 293T cells stably expressing A3F from the conditions described in G was determined. All data shown as means ± SD from ≥3 independent experiments; ANOVA was used to analyze the differences (* *p* < 0.05, ** *p* < 0.01, **** *p* < 0.0001, ns *p* > 0.05).

The same amount of virus (based on p24 ELISA) collected from supernatants of cells treated with each compound or control were pelleted, lysed, and probed for A3G or A3F on immunoblots. Virions produced in the presence of the NU compounds contained more A3G (Figure 5A) or A3F (Figure 5C) protein signal, relative to an equivalent amount of virions from DMSO-exposed cultures.

Virus-containing supernatants normalized for p24 were also used to infect TZM-bl reporter cells. One day post-infection, viral infectivity was measured by determining the luciferase activity of TZM-bl cell lysates. Virus from cultures co-transfected with Vif+ NL4.3 and A3G or A3F followed by treatment with DMSO had about a 20% reduction of viral infectivity compared to virus from cultures without co-transfected A3G (Figure 5B, DMSO) or A3F (Figure 5D, DMSO). Virus-containing supernatants from cultures treated with NU-52 or NU-302 after co-transfection with Vif+ NL4.3 and either A3G or A3F showed significant reductions of Vif+ virus infectivity, each relative to virus from co-transfected cells treated with DMSO. This was a reduction of 60% or more for A3G (Figure 5B, NU-52 or NU-302) and 40% or more for A3F (Figure 5D, NU-52 or NU-302); each relative to DMSO. In contrast, Vif+ or Vif-null viruses produced in the presence of either NU-52, NU-302, or NU-611 from control cells lacking any A3 expression did not differ in relative infectivity from viruses from DMSO-treated cells (Supplementary Figure S3). These latter controls are consistent with the three NU compounds acting by increasing cell and virion A3 content.

**Figure 6:**
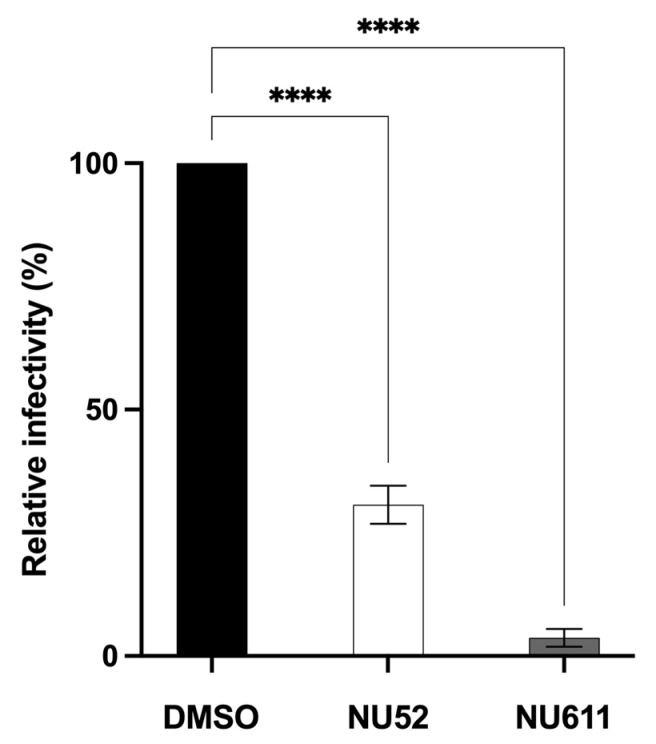
NU-611 enhances A3G-mediated reduction in HIV virion infectivity more than NU-52. 293T cells stably expressing A3G (293T IP/G cells) were transfected with Vif+ NL4.3 and then treated with either DMSO control or 15 μM each of either NU-52 or -611. DMSO was plotted as 100% relative infectivity. Data from 8 independent experiments are expressed as means ± SD; ANOVA was used to analyze the differences, **** *p* < 0.0001.

Virion A3 incorporation and relative infectivity were also tested in supernatants from Vif+ HIV transfections of 293T cell lines stably expressing A3G and A3F not fused to luciferase. This assessed if NU compound effects would be similar to those seen with the A3G-Luc fusion protein. These cell lines had been transduced with a retroviral vector encoding either A3G (pMSCV-I/Puro A3G) or A3F (pMSCV-I/Puro A3F) and selected in puromycin. The stably A3-expressing cell lines (293T IP/G with A3G and 293T IP/F with A3F) were transfected with the Vif+ NL4.3 expression plasmid. One day post-transfection, DMSO, NU-52, or NU-302 were added with fresh media. An additional 24 h later, supernatants were collected and normalized by HIV p24 ELISA. Again, treatment with NU-52 and NU-302 increased the incorporation of A3G (Figure 5E) or A3F (Figure 5G) into pelleted virus and reduced virus infectivity (Figure 5F,H), relative to DMSO. When NU-52 was compared to NU-611 in 293T IP/G cells, NU-611 increased virion A3G content and decreased A3G-mediated HIV virion infectivity more than NU-52 (Figure 6).

**Figure 7:**
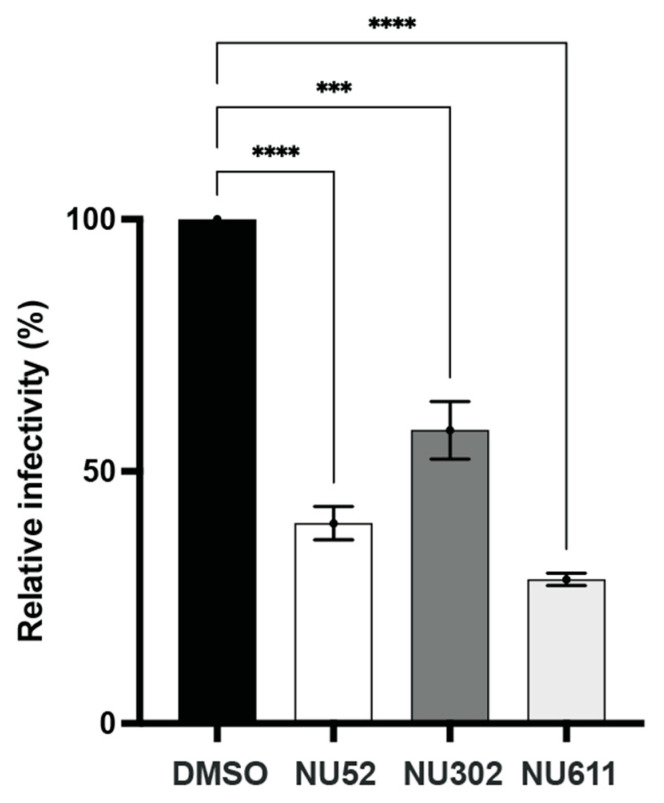
NU compounds decrease infectivity of Vif+ HIV produced by H9 cells expressing endogenous A3G and A3F after infection. After Vif+ NL4.3 infection, H9 cells were treated with a compound (NU-52, -302, or -611, each at 10 μM) or control (DMSO). TZM-bl cells were infected with p24-normalized H9 culture supernatants and relative infectivity quantified by luciferase activity of TZM-bl cell lysates. Infectivity of virus from DMSO control is plotted as 100%. All data expressed as means ± SD from ≥3 independent experiments; ANOVA was used to analyze the differences (*** *p* < 0.001, **** *p* < 0.0001, ns *p* > 0.05).

We also tested the effect of NU compound-mediated inhibition on Vif+ HIV infectivity by infecting a T-cell line that expresses endogenous A3G and A3F with Vif+ HIV. H9 cells were infected with Vif+ NL4.3 viral particles. The next day, NU-52, NU-302, NU-611, or DMSO control were added for 24 h. Supernatants were collected and the virus quantified by HIV p24 ELISA. Equal amounts of viral particles were used to infect TZM-bl cells. After 2 days, TZM-bl luciferase activity was measured. The infectivity of virus from Vif+ NL4.3-infected, NU compound-treated H9 cells was reduced significantly compared to that of virus from DMSO-treated cells (Figure 7).

### 3.4. NU Compounds Increase A3G Protein Stability in Uninfected Cells

We next evaluated a mechanism for the increased cellular levels of A3G and -F proteins following treatment with the NU compounds, starting with the assessment of transcription. The control cell line expressing only luciferase did not have increased luciferase expression after treatment with NU-52, -302, or -611, indicating no activation of the promoter present in the A3G-Luc fusion protein. We also evaluated the transcriptional activation of the A3s in 293T cells transiently transfected with A3G or A3F expression plasmids, as well as in an endogenous A3G- and A3F-expressing T-cell line (CEM). Each of these cells was treated with NU-52, -302, -611, or DMSO. After 24 h, no difference was seen in A3G or A3F RNA levels in cells exposed to NU compounds versus DMSO, as measured by RT-qPCR.

**Figure 8:**
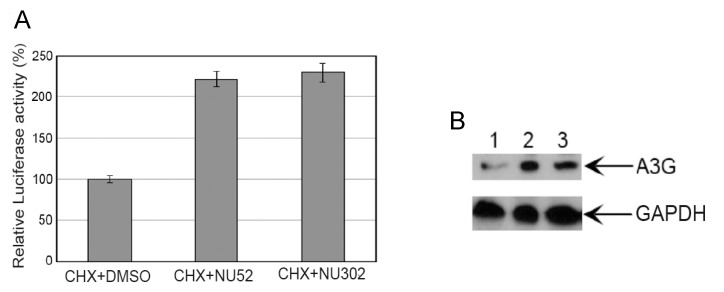
Stability of A3G and A3F in the presence of protein synthesis-blocking cycloheximide (CHX) increases with treatment with NU-52 or -302 at 10 μM concentration. (**A**) Relative luciferase activities are shown from the cell line stably expressing intermediate levels of A3G-Luc (A3G-Luc #6) after treatment with either CHX+DMSO, CHX+NU-52, or CHX+NU-302. (**B**) Immunoblots from 293T cells transfected with A3G expression plasmid are shown after treatment with either CHX and DMSO (lane 1), CHX plus NU-52 (lane 2), or CHX plus NU-302 (lane 3).

Because of the lack of evidence of changes in the transcriptional regulation, we next evaluated the post-translational stability of A3G protein by treating A3G-Luc stably expressing clone #6 (intermediate A3G expression level) with cycloheximide (CHX) alone or a combination of CHX and either NU-52 or NU-302. CHX inhibits protein biosynthesis, so higher levels of luciferase activity were hypothesized if the compounds decreased post-translational degradation of A3G-Luc. After overnight treatment with CHX plus either NU-52 or NU-302, luciferase activity was higher than with CHX plus DMSO (Figure 8A). This indicates that NU-52 and NU-302 increase the stability of the A3G-Luc protein post-translationally, independently of Vif. The same experimental approach was applied to 293T cells transfected with an A3G expression plasmid. More A3G protein was detected with CHX plus NU-52 or NU-302 by immunoblotting, relative to CHX plus DMSO (Figure 8B).

### 3.5. Compounds Block VHL-Mediated Degradation of A3G and -F

Previous work has demonstrated that CRL^pVHL^ function caused proteasomal degradation of all A3 proteins, although A3G was less efficiently degraded than A3B [36]. Since the NU compounds increase protein levels of A3F and A3G in the absence of HIV Vif, we tested whether the compounds inhibited VHL-mediated degradation of A3G and A3F. 293T cells were transiently co-transfected with pVHL and either HA-tagged A3G or A3F. NU-52, NU-611, or DMSO were added 4 h after transfection. One day post-transfection, cell lysates were collected and analyzed by immunoblotting.

**Figure 9:**
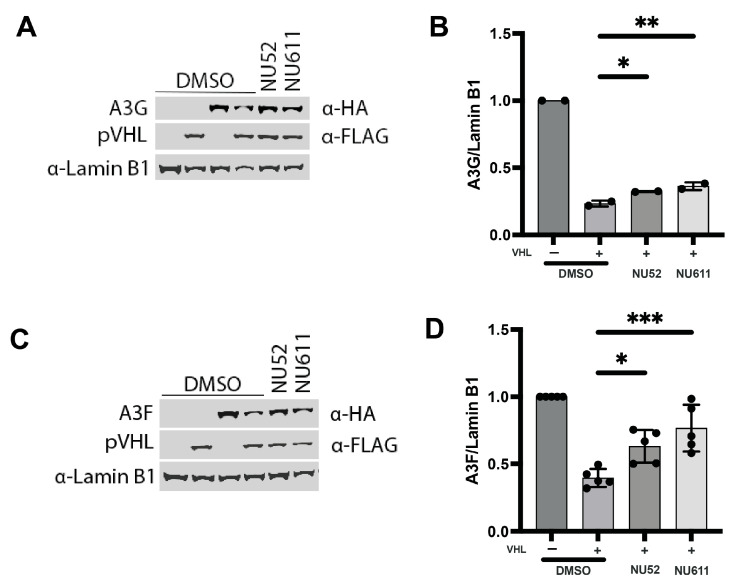
NU-52 and -611 diminish pVHL-mediated degradation of A3G or -F. (**A**) Immunoblots from cell lysates of 293T cells transfected with pVHL and A3G are shown. (**B**) Band densities of immunoblots depicted in A are quantified. (**C**) Immunoblots from cell lysates of 293T cells transfected with pVHL along with A3F are shown. (**D**) Band densities of immunoblots depicted in C are quantified. Mean ± SD for at least 2 independent immunoblots are shown. ANOVA was used to analyze the differences (* *p* < 0.05, ** *p* < 0.01, *** *p* < 0.001).

A3G or A3F protein levels were reduced with co-transfection of pVHL in the DMSO control (Figure 9). However, treatment with NU-52 or NU-611 increased A3G (Figure 9A,B) or A3F (Figure 9C,D) protein levels compared to the DMSO control. This indicates that inhibition of pVHL-mediated degradation contributes to the increased levels of A3G or A3F caused by NU-52 and NU-611.

## 4. Discussion

Extensive research has shown that A3G blocks infectivity of HIV produced in the absence of Vif-mediated proteasomal degradation [18]. Some evidence also suggests that the presence of an increased amount of cellular A3G protein prior to infection better decreases wild-type, Vif+ HIV-1 infectivity [27,28,40], albeit not completely. Here, we sought to optimize a compound, IMB-26, that was previously identified to boost A3G levels by antagonizing Vif [29]. We designed and synthesized less cytotoxic analogs of IMB-26 that increased A3 levels and their anti-HIV function. The novel compounds described in detail here (NU-52, NU-302, and NU-611) increased cellular and HIV virion-incorporated A3G or A3F, whether Vif was present in the virus-producing cell or not. The increased incorporation of A3G and A3F into budding virions decreased their infectivity, even in the presence of functional Vif. NU-611 decreased HIV-1 viral infectivity more strongly than NU-52 or NU-302 and had less cytotoxicity. None of the analogs synthesized here boosted A3G or A3F solely in the presence of Vif, suggesting a mechanism other than antagonism of Vif function (Supplementary Table S1). NU-52, NU-302, and NU-611 did not alter the infectivity of virions produced from cells lacking any A3 expression, consistent with acting by increasing cell and virion A3 (Supplementary Figure S3).

How these compounds boosted levels of A3G and -F proteins and diminished virion infectivity was studied in both uninfected and infected cells. The compounds did not induce transcriptional activation of the promoter driving A3G-Luc fusion protein expression. Lack of transcriptional activation was also seen for two other promoters: those in the A3G or -F expression vectors (pcDNA3-A3F/-G) used in the co-transfection experiments with HIV expression plasmids and the endogenous A3G promoter in CEM cells. Moreover, CHX treatment of A3G-Luc cells and cells stably expressing unfused A3G demonstrated that NU-52 and NU-302 each increased the post-translational stability of A3G in the absence of Vif. These data strongly indicated that these compounds impair cell-intrinsic mechanisms of A3G and A3F degradation, rather than the viral Vif-mediated mechanism of A3G and A3F degradation. NU-52 and NU-611 each inhibited a specific cell-encoded regulatory pathway we had described previously: pVHL-mediated degradation of A3G and -F [36]. While significant, this effect did not completely restore levels of the A3s to those of the control and may not account for all the effects of the compounds to decrease A3 degradation. Future work will focus on identifying other cellular factors that degrade A3G and A3F without functional Vif and studying whether the NU compounds also block degradation by other cellular factors. Unlike another compound, RN-18, identified before to inhibit A3G degradation by the viral protein Vif [30,31], results here suggest limited potential for emergence of Vif mutation-mediated viral escape from effects of NU compounds since results here indicate that their mechanism of action is dependent on cellular machinery.

Given the significant effects of the NU compounds on cellular A3 levels, additional research is suggested. Effects of increasing A3G and A3F should be characterized in uninfected T cells and macrophages, including evaluating potential effects on transposable elements or other RNAs. In addition to characterizing the mechanisms of action in more detail in future work and assessing effects on uninfected immune cells, study of animal pharmacology and toxicology for these compounds is needed. Preliminary results showed that a single intraperitoneal (IP) dose (up to 200 mg/kg) of NU-611 (the least cytotoxic of the NU compounds) was well tolerated in immunocompetent CD-1 mice. (In contrast, a single dose of NU-52 was tolerated up to 75 mg/kg.) Twice weekly IP NU-611 (at 5 mg/kg) was also well tolerated over 3 weeks in a different strain of humanized mice [41,42,43]. Anti-HIV effects were seen in the HIV-infected, ART-suppressed humanized mice administered short-term NU-611 when ART was stopped [44].

In summary, we have established stable cell lines and processes that identified compounds boosting A3G and A3F protein levels in HIV virions by countering cell-intrinsic A3G and A3F degradation. The compounds also increased A3G in resting CD4+ T cells in the absence of Vif. The processes and cell lines used here can be adapted for higher throughput use, potentially enabling accelerated optimization of other leads or screening of chemical libraries for A3G and A3F boosting. The novel compounds described here are also a starting point for the development of therapeutics that decrease the infectivity of virions reactivated from resting CD4+ T cells harboring latent proviruses after stopping ART. Such an agent could be an adjunctive component of a therapeutic strategy aiming for ART-free remission of HIV that can be administered when ART is stopped and that minimizes risk of escape mutation selection in Vif.

## 5. Patents

US patent 9,688,637 B2 issued 27 June 2017.

## Data Availability

The original contributions presented in this study are included in the article/Supplementary Material. Further inquiries can be directed to the corresponding author.

## Supplementary Materials

The following supporting information can be downloaded at: https://www.mdpi.com/article/10.3390/v17040514/s1, Table S1: Analogs synthesized and evaluated; Figure S1. Diagram of syntheses of NU compounds; Figure S2. Effects of IMB-26 and NU-52 on A3G; Figure S3. Effects of NU compounds on virus infectivity.

## Abbreviations

The following abbreviations are used in this manuscript:

HIV	Human Immunodeficiency Virus
APOBEC3	Apolipoprotein B mRNA-editing enzyme catalytic polypeptide-like 3
RLU	Relative Luciferase Unit
VHL	Von Hippel Lindau
CHX	Cycloheximide
YFP	Yellow Fluorescent Protein
