# Supplementary material for: Design and Characterization of Inhibitors of Cell-Mediated Degradation of APOBEC3G That Decrease HIV-1 Infectivity
Source: Viruses. 2025 Apr 1;17(4):514. doi: 10.3390/v17040514 (PMC12031279; doi:10.3390/v17040514)
Supplement: Supplementary file 1 [file viruses-17-00514-s001.zip › viruses-3563058-supplementary.pdf]

Supplementary Materials

Table S1. Analogs modified from IMB-26 and evaluated

**Structure** (methyl groups depicted as lines)	**NU-# or Compound ID #** (used at 10uM, unless otherwise indicated)	**A3G-Luc Activity (% of control)**	**Vif/A3G-Luc Activity (% of control)**
Halogenated compounds			
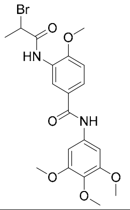	IMB-26 (5uM)	49	75
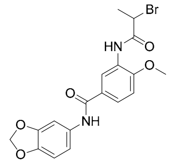	54051	57	55
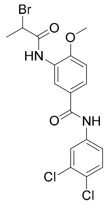	54053	172	142
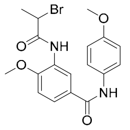	54055 (5uM)	56	74
Des-bromo compounds			
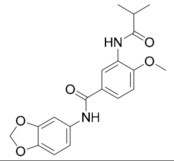	54050 (30uM)	168	145
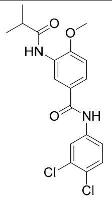	54052	178	155
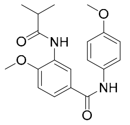	54054 (30uM)	137	116

**Structure** (methyl groups depicted as lines)	**NU-# or Compound ID #** (used at 10uM, unless otherwise indicated)	**A3G-Luc Activity (% of control)**	**Vif/A3G-Luc Activity (% of control)**
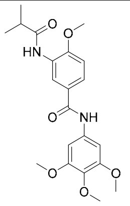	54056 (30uM)	114	93
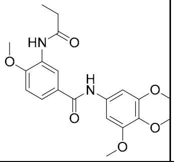	54057 (30uM)	87	74
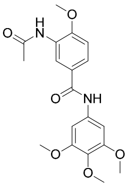	54058 (30uM)	123	93
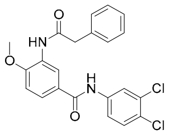	125308	92	113
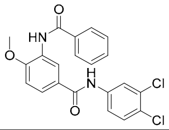	125309	100	94
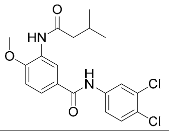	NU-52 (formerly 125452)	178	135
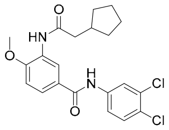	125453	169	95
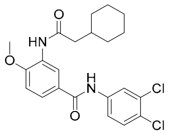	125454	86	84
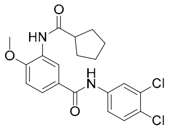	125455	188	131
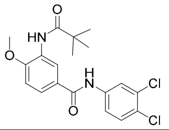	125458	189	123

**Structure** (methyl groups depicted as lines)	**NU-# or Compound ID #** (used at 10uM, unless otherwise indicated)	**A3G-Luc Activity (% of control)**	**Vif/A3G-Luc Activity (% of control)**
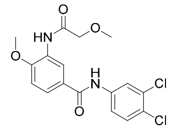	125462	282	170
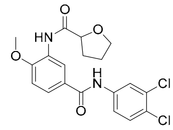	125464	288	170
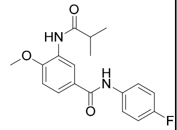	125564	162	102
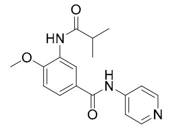	125565	66	55
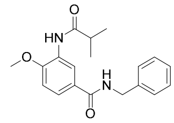	125566	106	80
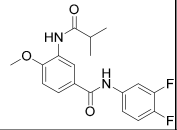	125571	116	104
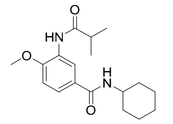	125573	117	80
Heteroatom-containing des-bromo compounds			
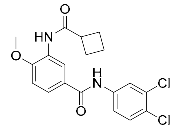	NU-302 (formerly 125302)	196	167
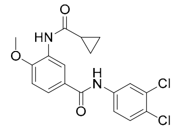	125304	124	138
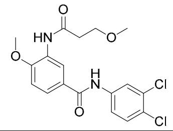	125463	287	178
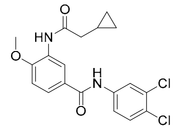	125466	318	202

**Structure** (methyl groups depicted as lines)	**NU-# or Compound ID #** (used at 10uM, unless otherwise indicated)	**A3G-Luc Activity (% of control)**	**Vif/A3G-Luc Activity (% of control)**
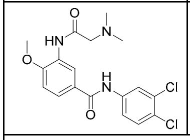	125471	238	175
Des-bromo compounds with an amide replaced by a carbamate, sulfonamide, or urea			
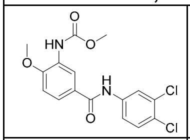	125460	254	119
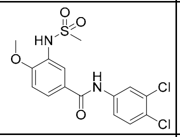	125467	194	146
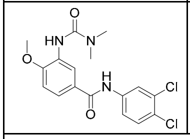	125472	238	146
Ether-containing des-bromo compounds			
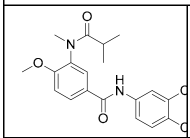	NU-611 (formerly 125611)	197	175
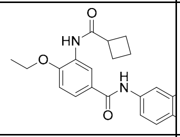	125612	202	161
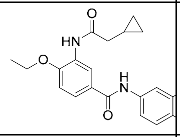	125613	186	158
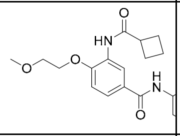	125616	171	152
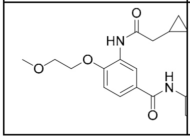	125617	191	180

Supplementary Figures

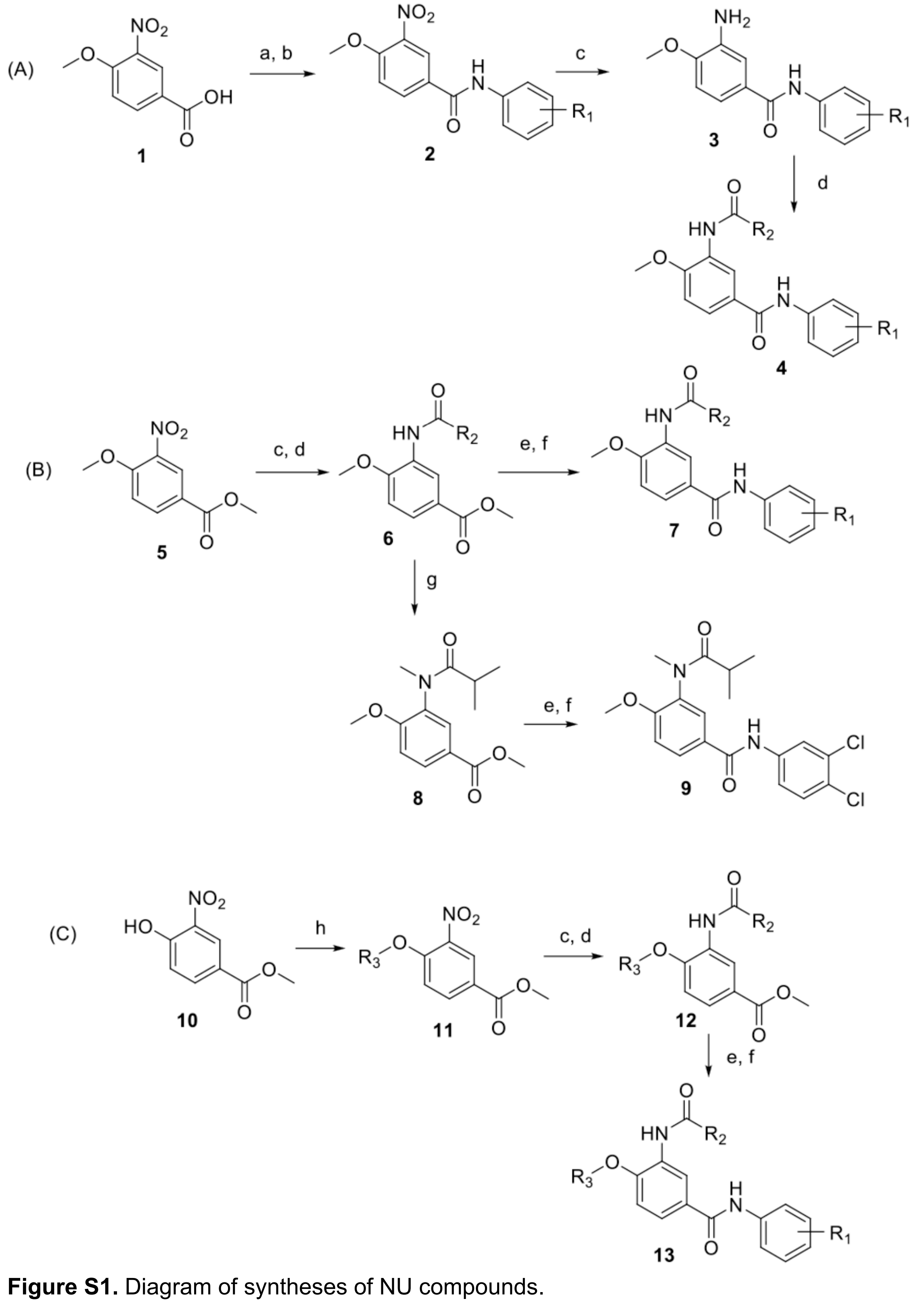

Figure S1. Diagram of syntheses of NU compounds.

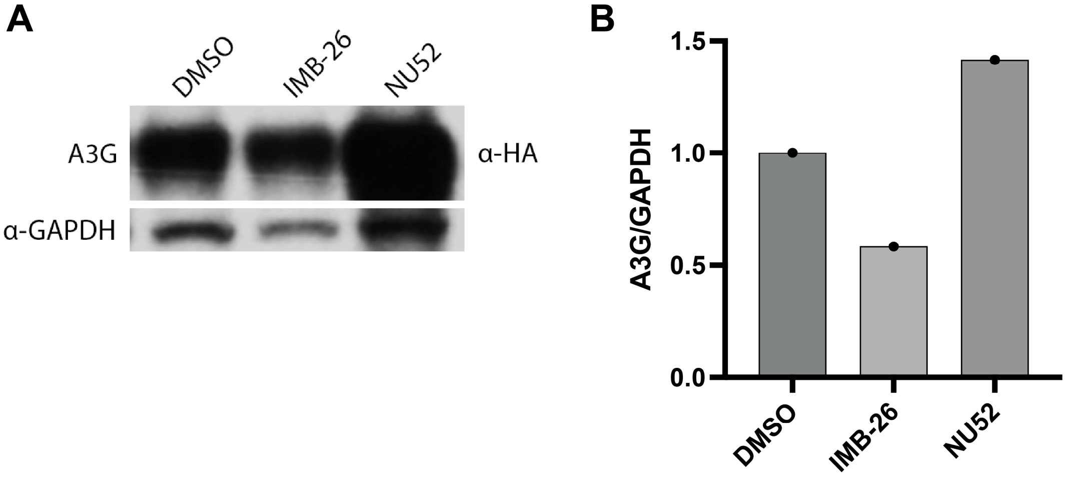

Figure S2. Effects of IMB-26 and NU-52 on A3G. **A.** Treatment of 293T cells transiently transfected with A3G expression plasmid with NU52 increased A3G, relative to DMSO. **B**. Quantification of band densities in A.

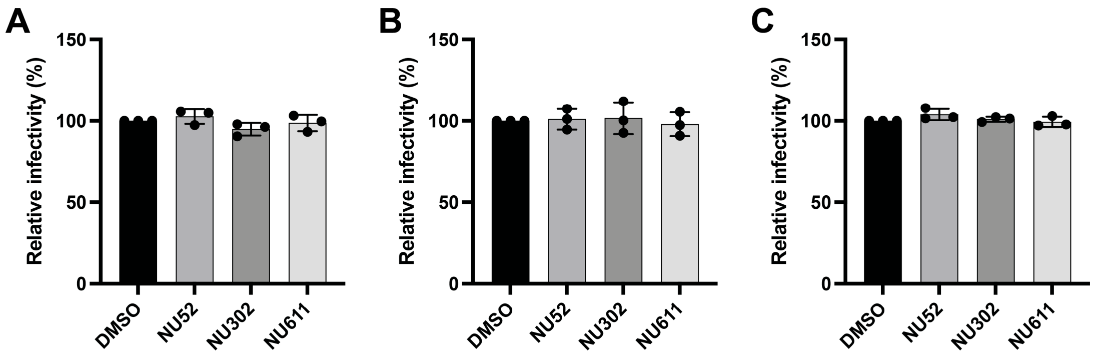

Figure S3. Effects of NU compounds on virus infectivity. NU compounds do not alter relative infectivity on TZM-bl cells of Vif-null or Vif+ NL4.3 supernatants produced from cell lines lacking expression of A3s. The same methods were used in experiments shown in each panel. Supernatants were normalized using p24 ELISA and TZM-bl cells were infected with uniform amounts of p24. RLU was read 24 hours after infection and normalized to DMSO control. **A.** Vif-null NL4.3 produced from 293T cells in the presence of either NU-52, NU-302, or NU-611 did not have a difference in infectivity from supernatants produced from DMSO-treated cells. **B.** Vif+ NL4.3 produced from 293T cells in the presence of either NU-52, NU-302, or NU-611 did not have a difference in infectivity from supernatants produced from DMSO-treated cells. **C.** Vif+ NL4.3 produced from 293T I/Puro (empty vector control) cells in the presence of either NU-52, NU-302, or NU-611 did not change infectivity, relative to supernatants from DMSO-treated cells.
